# The novel HS-mimetic, Tet-29, regulates immune cell trafficking across barriers of the CNS during inflammation

**DOI:** 10.1186/s12974-023-02925-4

**Published:** 2023-11-01

**Authors:** Tessa Peck, Connor Davis, Georgia Lenihan-Geels, Maddie Griffiths, Sam Spijkers-Shaw, Olga V. Zubkova, Anne Camille La Flamme

**Affiliations:** 1 School of Biological Sciences, Victoria University of Wellington, Wellington, New Zealand; 2 Centre for Biodiscovery Wellington, Victoria University of Wellington, Wellington, New Zealand; 3 Ferrier Research Institute, Victoria University of Wellington, Wellington, New Zealand; 4 Malaghan Institute of Medical Research, Wellington, New Zealand

**Keywords:** EAE, MS, HS, HSPG, Neuroinflammation, Migration, Tet-29, BBB, Choroid plexus

## Abstract

**Background:**

Disruption of the extracellular matrix at the blood–brain barrier (BBB) underpins neuroinflammation in multiple sclerosis (MS). The degradation of extracellular matrix components, such as heparan sulfate (HS) proteoglycans, can be prevented by treatment with HS-mimetics through their ability to inhibit the enzyme heparanase. The heparanase-inhibiting ability of our small dendrimer HS-mimetics has been investigated in various cancers but their efficacy in neuroinflammatory models has not been evaluated. This study investigates the use of a novel HS-mimetic, Tet-29, in an animal model of MS.

**Methods:**

Neuroinflammation was induced in mice by experimental autoimmune encephalomyelitis, a murine model of MS. In addition, the BBB and choroid plexus were modelled in vitro using transmigration assays, and migration of immune cells in vivo and in vitro was quantified by flow cytometry.

**Results:**

We found that Tet-29 significantly reduced lymphocyte accumulation in the central nervous system which, in turn, decreased disease severity in experimental autoimmune encephalomyelitis. The disease-modifying effect of Tet-29 was associated with a rescue of BBB integrity, as well as inhibition of activated lymphocyte migration across the BBB and choroid plexus in transwell models. In contrast, Tet-29 did not significantly impair in vivo or in vitro steady state-trafficking under homeostatic conditions.

**Conclusions:**

Together these results suggest that Tet-29 modulates, rather than abolishes, trafficking across central nervous system barriers.

**Supplementary Information:**

The online version contains supplementary material available at 10.1186/s12974-023-02925-4.

## Background

Disruption of the blood–brain barrier (BBB) occurs in many neuroinflammatory disorders including Alzheimer’s disease, multiple sclerosis (MS), and stroke [1]. As such, there is a growing need for therapeutics to maintain BBB integrity. A key regulator of BBB structure and organisation is heparan sulfate (HS) [2]. HS is a highly sulphated glycosaminoglycan that, when attached to a protein core to form a HS-proteoglycan (HSPG), serves a variety of critical functions in cell signalling, migration, and regulation [3]. HSPGs are expressed ubiquitously at cell surfaces and in the extracellular matrix (ECM) where they can be incorporated into the basal lamina, and due to its high negative charge, HS can bind chemokines to form chemotactic gradients. These activities are important for regulating the migration of immune cells in response to chemotactic signals and through ECM barriers such as blood vessel-associated basement membranes, including those in the BBB. These functions of HS make it an attractive therapeutic target to prevent the migration of pathogenic cells such as metastatic cancer cells or autoreactive neuroinflammatory immune cells [4].

Heparanase (HPSE), an endoglycosidase, is the only enzyme capable of cleaving HS and thus is an important regulator of HS-mediated functions [5]. It is expressed by several cell types including tumour cells, T cells, and endothelial cells, and via its ability to cleave HS, HPSE participates in ECM breakdown and remodelling [6]. While the role of HPSE in cancer, where it promotes tumour metastasis, is well described [7], its involvement in neuroinflammatory diseases such as MS is less well understood. However, it has been implicated in numerous autoimmune pathologies including those characterised using experimental autoimmune encephalomyelitis (EAE), an animal model of MS [8–12].

Previous studies have shown that HPSE inhibitors, including purified HS, heparin, or HS mimetics, are effective in EAE [13–16]. These studies reported that inhibiting HPSE reduced disease scores as well as the infiltration of immune cells into the central nervous system (CNS), suggesting that HPSE inhibition is a promising therapeutic strategy for MS [17, 18]. Although targeting HPSE therapeutically is an attractive idea, there are two key obstacles to overcome. First, HS is chemically complex and has enormous structural diversity, which affects the cost effectiveness of its synthesis. Second, many HPSE inhibitors show high anti-coagulant activity. For example, pixatimod (PG545) and PI-88 are HPSE inhibitors developed for use in cancer that, like heparin, have anticoagulant activity [19]. Therefore, novel HPSE inhibitors should be simple and cost-effective to synthesise and have no anti-coagulant activity to be clinically useful.

**Fig. 1:**
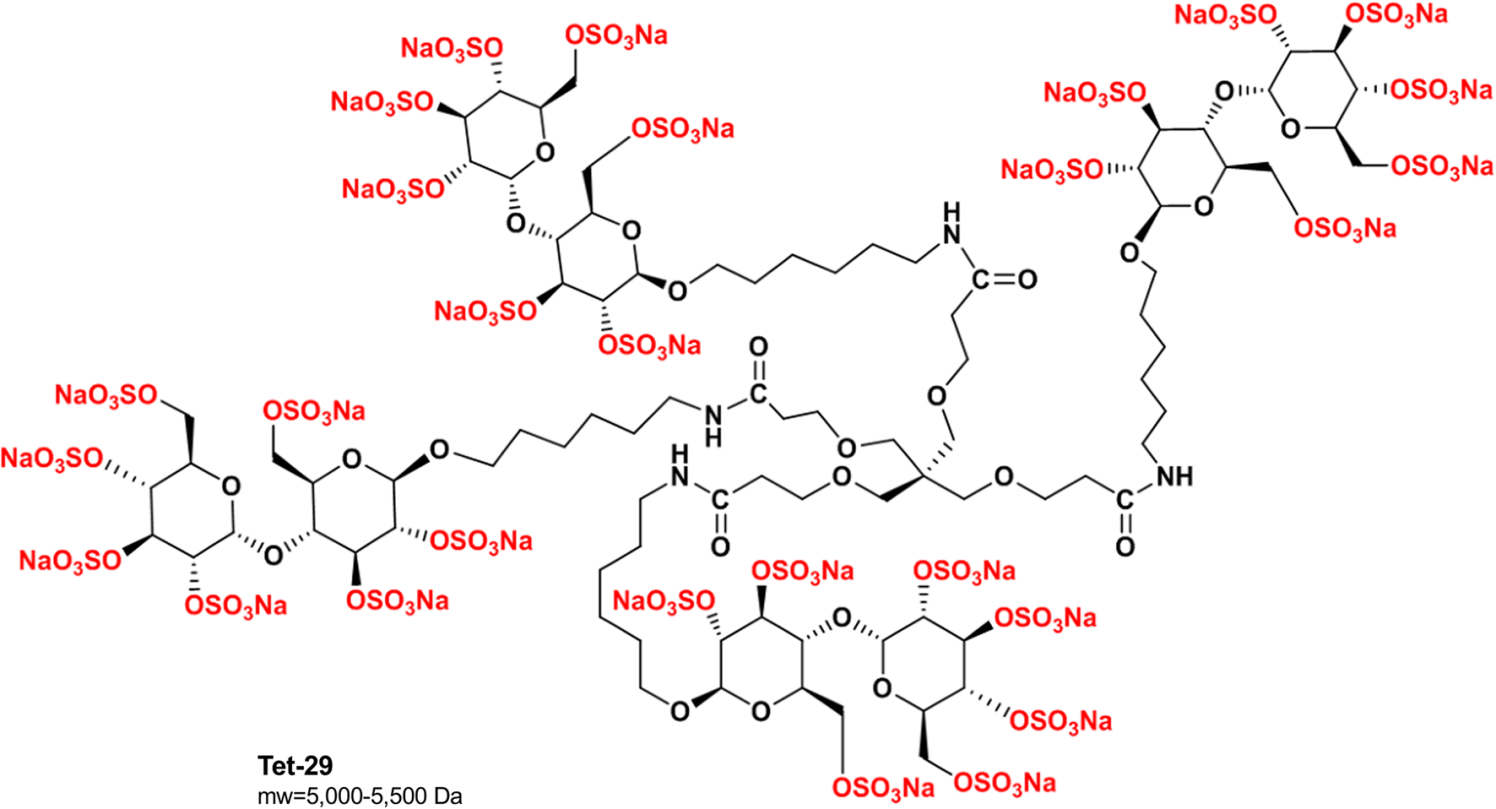
The chemical structure of Tet-29. Tet-29 comprises of a four-armed dendritic core capped with highly sulfated maltose-derived saccharides

We have developed a bio-inspired HS mimetic, Tet-29 (Fig. 1), that potently inhibits HPSE without anti-coagulant activity [20]. Tet-29 has a small dendrimer structure, unlike the linear oligosaccharide structure of native HS, PG545, and PI-88 (Additional file 1: Fig S1). This dendrimer structure relies on the polyvalent interactions at multiple binding sites to create a synergistic clustering effect [21]. Here, we investigated the ability of Tet-29 to reduce the infiltration of activated immune cells into the CNS using the EAE model of neuroinflammatory MS.

## Methods

### Compounds for treatment

Tet-29 (Fig. 1), BODIPY-labelled Tet-29 (BDP-Tet-29) and unsulfated BDP-Tet-29 were provided by Dr Olga Zubkova and Sam Spijkers-Shaw from the Ferrier Research Institute (Victoria University of Wellington (VUW), Wellington, NZ). PI-88 (Additional file 1: Fig S1) was kindly donated by Professor Chris Parish (John Curtin School of Medical Research, Canberra, Australia). The anti-mouse/human VLA-4 (PS/2; CD49d) and rat IgG2b anti-keyhole limpet hemocyanin (LTF-2) antibodies were purchased from Bio X Cell (Lebanon, NH, USA). All compounds were reconstituted in sterile phosphate-buffered saline (PBS; i.e. vehicle) and administered to mice via intraperitoneal (i.p.) injection.

### Mice

Female C57Bl/6J mice were bred and held in a temperature- and humidity-controlled environment at the VUW Animal Research Facility or the Biomedical Research Unit at the Malaghan Institute of Medical Research (Wellington, NZ). All housing and experimental procedures were approved by the VUW Animal Ethics Committee under the approvals AEC25295 and AEC29122.

### EAE induction

EAE was induced in 8–12-week old mice. Briefly, mice were immunised with 50 µg of MOG_35–55_ (Genscript, Piscataway, NJ, USA) in complete Freund’s adjuvant (Sigma, St. Louis, MO, USA) containing 500 µg/mouse heat-killed *Mycobacterium* (Difco Laboratories, Franklin Lakes, NJ, USA) by subcutaneous (s.c.) injection in the rear flanks. Concurrently, mice received a 200 ng dose of pertussis toxin (PTX; List Labs, Campbell, CA, USA) by i.p. injection. Two days later, mice were treated with a second 200 ng PTX dose. Following immunisation, mice were weighed and scored daily by a single unblinded observer. EAE severity was scored as follows; 0 = not affected, 0.5 = tail weakness or distal tail paralysis, 1 = significant tail weakness or half tail paralysis, 2 = full tail paralysis, 3 = one hind limb paralysis or severe weakness in both hindlimbs, 4 = full hindlimb paralysis, 5 = moribund. Mice were considered recovered from disease when they reached a score of ≤ 0.05 after their initial peak in disease. Relapse was defined as an increase in disease score by ≥ 1 for ≥ 2 days after the initial peak in disease.

### Primary cell isolation into single-cell suspension

Tissues were collected after CO_2_ asphyxiation and processed into single-cell suspensions. Animals were perfused with 20 mL of PBS before brains and spinal cords were collected. Spinal cords were minced with a scalpel then digested in a 2.4 mg/mL type II collagenase solution (Gibco, Thermo Fisher Scientific, Waltham, MA, USA) for 30 min at 37 °C under constant agitation. Both brains and digested spinal cords were processed into single-cell suspensions by forced passage through a 70 µm cell strainer. CNS tissues were centrifuged for 5 min at 760 × g before resuspension in a 37% Percoll (Sigma-Aldrich) solution. A Percoll gradient was created by centrifuging samples for 30 min at a low acceleration and no deceleration. Myelin was removed from the top of the gradient before cells were washed, pelleted by centrifugation, and resuspended in flow cytometry staining (FACs) buffer containing 2% foetal calf serum (FCS, Gibco) and 0.1% sodium azide (1 M).

Blood was collected in ethylenediaminetetraacetic acid (EDTA)-coated tubes to prevent coagulation. Samples were incubated in red blood cell lysis buffer (Sigma-Aldrich) for 2 min before being rinsed with wash buffer and centrifuged for 5 min at 760 × g. Spleens were collected and mashed through a 70 µm cell strainer before the cell lysis step was carried out. After satisfactory lysis, splenocytes and blood cells were rinsed, centrifuged, and resuspended in FACs buffer.

### Flow cytometry

Single-cell suspensions in FACs buffer were incubated in Fc block (2.4G2, BD Biosciences, Franklin Lakes, NJ, USA) for 15 min then samples were washed in FACs buffer and centrifuged at 400 × g for 4 min. Cells were then incubated in a solution containing fluorescently labelled antibodies for 30 min in the dark on ice. Following staining, flow cytometry was performed on a BD FACS Canto II (BD Biosciences) or a LSRFortessa™ (BD Biosciences). Analysis was performed on FlowJo software (BD Biosciences) using the gating strategy outlined in Additional file 2: Fig S2.

The following antibodies were used for the detection and phenotyping of immune cells: CD45-BV510 (30-F11; Biolegend, San Diego, CA, USA), CD3-APC (17A2; Biolegend), B220-APC-Cy7 (RA3-6B2; Biolegend), CD4-BV421 (RM4-5; Biolegend), CD8a-PerCP-Cy5.5 (53–6.7; Biolegend), CD62L-FITC (MEL-14; Biolegend), CD44-PE (IM7; Biolegend), CD11b-PE-Cy7 (M1/70; Biolegend), Ly6G-APC (1A8; Biolegend), Ly6C-PE (HK1.4; Biolegend), and CD49d-AF488 (R1-2; Biolegend).

### Immunohistochemistry

Brains were fixed in 4% paraformaldehyde then cryoprotected in a 30% sucrose (Sigma-Aldrich) solution for 48 h. Samples were snap frozen in PolyFreeze (Sigma-Aldrich) and cryo-sectioned into 20 µm thick sections on a Leica CM3050 cryotome (Leica Microsystem, Wetzlar, Germany). Sections were heated to 70 °C in Tris–EDTA (Sigma-Aldrich) buffer for 10 min and then incubated in 1 mg/mL sodium borohydride (Sigma-Aldrich) quench for 30 min. Following quenching, sections were blocked using 5% donkey serum (Sigma-Aldrich) in PBS with 0.05% Tween20 (Sigma-Aldrich) for 2 h at room temperature. Primary antibodies: rabbit anti-mouse CD31 (1:200, cat. # ab182981, Abcam, Cambridge, UK), goat anti-mouse Albumin (1:200, cat. # ab19194, Abcam), rat anti-mouse VCAM-1/CD106 (1:200, cat. # 105702, Biolegend), rat anti-mouse ICAM-1 (1:200, cat. # 116102, Biolegend), and rabbit anti-mouse Collagen IV (1:200, cat. # ab19808, Abcam) were added to blocking buffer (PBST and 5% donkey serum; Sigma-Aldrich) and then applied to sections overnight at 4 °C. Slides were then washed in PBST and secondary antibodies: anti-goat Alexa Fluor 488 (1:1000, cat. # ab150129, Abcam), anti-rabbit Alexa Fluor 647 (1:800, cat. # ab150075, Abcam), anti-rat Alexa Fluor 568 (1:800, cat. # ab175475, Abcam) were added to PBS and incubated for 2 h at room temperature. After washing off the secondary antibodies with PBS-Tween20, ethanol washed coverslips were mounted using anti-fade glycerol DAPI containing mounting medium (Abcam).

Images were obtained using an Olympus FV3000 laser scanning confocal microscope using the 20× and 40× air objectives (Olympus, Lower Hutt, New Zealand) by z-series imaging with a 0.60 µm step size. Images were processed using ImageJ [22] features “Remove Bright Outliers” and Median filter. Cell Profiler [23] was used to obtain measurements of area using the following modules: “ColourToGray”, “Threshold”, and “MeasureImageAreaOccupied.”

### Cell culture

The transformed C57BL/6 *Mus musculus* epithelial choroid plexus cell (ECPC-4) cell line (RRID: CVCL_4836) was provided by the RIKEN Bioresource Centre through the National BioResource Project of the AMED, Japan. ECPC-4 cells were cultured in Dulbecco’s modified Eagle medium (DMEM) supplemented with 10% FCS, 100 U/mL penicillin and 100 µg/mL streptomycin (all from Gibco) at 37 °C/5% CO_2_.

The murine cerebral brain endothelioma bEnd.3 cell line was obtained from the American Type Tissue Culture Collections (ATCC^®^ CRL™-2299™, Manassas, VA, USA). bEnd.3 cells were cultured in DMEM (ATCC 30-2002) supplemented with 10% FCS, 2 mM l-glutamine, 100 U/mL penicillin and 100 µg/mL streptomycin at 37 °C/5% CO_2_.

Splenocytes were isolated and seeded at 1 × 10^6^ cells/well in growth medium (DMEM plus 10% FCS, 100 U/mL penicillin, 100 µg/mL streptomycin, 10 mM HEPES, 2 mM l-glutamine, 50 μM 2-mercaptoethanol, and non-essential amino acids; Gibco) in a 96-well plate and incubated for 24 h at 37 °C with 1 µg/mL Concanavalin A (ConA, Sigma-Aldrich) or growth media alone. Treatment with Tet-29 was carried out in both conditions by supplementing the media with 60 µg/mL Tet-29.

### Seeding of monolayer cultures

bEnd.3 and ECPC-4 cells were seeded at 5 × 10^4^ cells per insert (CLS3422 for bEnd.3 and CLS3415 for ECPC-4; Coring Costar, Kennebunk, ME, USA) in growth media on the collagen-coated membranes. ECPC-4 cells were seeded on the basolateral side of the membrane and left to adhere for 12 h in the inverted position, and then reoriented in wells containing growth media. bEnd.3 cells were seeded on the apical side of the insert in wells containing growth media. Both cultures were left to form a confluent monolayer by incubating at 37 °C/5% CO_2_ for 48 h.

Growth media from the upper chamber of the transwell inserts was aspirated, and 1 × 10^6^ splenocytes were added to the upper chamber of confluent ECPC-4 or bEnd.3 transwell models. For Tet-29 treated conditions, monolayers were treated with 60 μg/mL Tet-29 for 24 h prior to the addition of splenocytes, and further treated with Tet-29 upon addition of splenocytes. Cultures were incubated at 37 °C/5% CO_2_ for 24 h. To assess the extent of migration across the monolayer, the upper and lower fractions were collected and prepared for flow cytometry. A migration index for each sample was calculated by dividing the number of migrating cells in the sample by the combined number of sample migrating cells plus the number of migrating cells in an unstimulated vehicle control.

### Statistical analysis

Statistical analysis was carried out using GraphPad Prism version 9.3.1 (GraphPad Software Inc., La Jolla, CA, USA). For parametric datasets, differences between means were established using unpaired Student’s t test, one-way, or two-way ANOVA, as indicated in figure captions. Multiple comparisons testing was carried out using the recommended post hoc test. All data are displayed as mean ± SEM, and differences between groups were considered statistically significant when the p value was ≤ 0.05.

## Results

### Tet-29 ameliorates disease and CNS infiltration in EAE

**Fig. 2:**
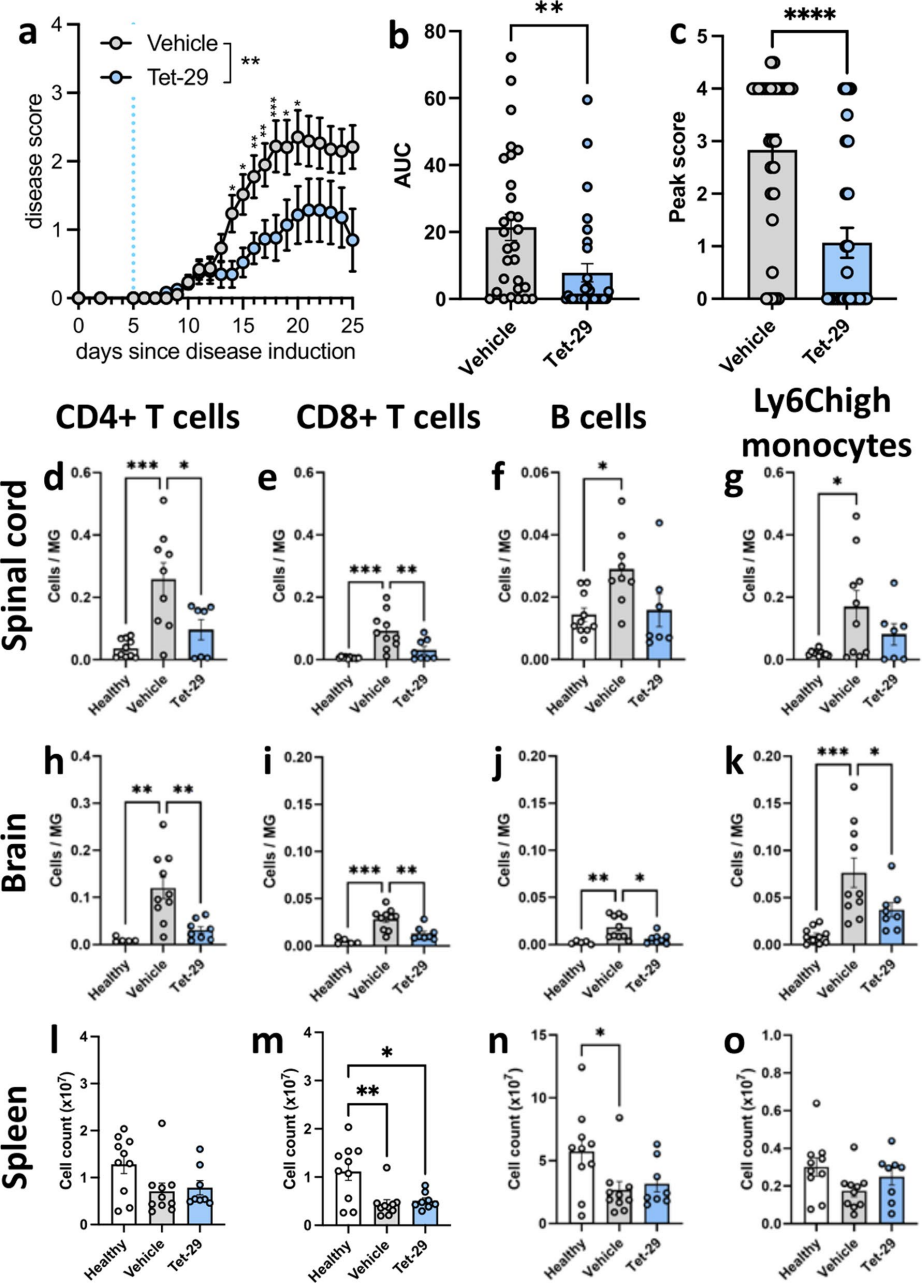
Tet-29 ameliorates disease by inhibiting CNS infiltration. Female C57BL/6J mice were immunised for EAE and treated daily with 30 mg/kg/day of Tet-29 (n = 24) or the equivalent volume of vehicle (n = 30) by i.p. injection. Healthy mice were treated daily with vehicle. Treatment began on day 5 after EAE induction and animals were sacrificed after 20 days of treatment. Mice were scored daily (**a**–**c**). Immune cell populations in the spinal cord (**d**–**g**), brain (**h**–**k**), and spleen (**l**–**o**) were analysed by flow cytometry. Data are pooled from 3 independent experiments and displayed as mean ± SEM, with n = 10 (healthy & vehicle) or 8 (Tet-29) per group. **a** An interaction between time and treatment group on scores was uncovered with **p < 0.0001 by mixed effects model and *p < 0.05, **p < 0.01, ***p < 0.001 by Sidak’s multiple comparison test. **b** **p < 0.01 by Student’s unpaired t-test. **c** ****p < 0.0001 by Mann–Whitney non-parametric test. **d**–**o** *p < 0.05, **p < 0.01, ***p < 0.001 by one-way ANOVA with Tukey’s multiple comparisons

First, we investigated the disease-modifying effect of Tet-29 delivered before the onset of disease as the compound was expected to inhibit the initial influx of immune cells into the CNS during the onset of EAE. A daily dose of 30 mg/kg/day (i.e. 600 μg/mouse/day) was used based upon a previous cancer therapy study from our group [20]. Vehicle (i.e. PBS)-treated EAE mice exhibited severe and unresolving disease whereas mice treated with daily Tet-29, from day 5 post EAE induction, had a significantly lower average disease score over the course of treatment (Fig. 2a). Additionally, Tet-29 treatment caused a significant reduction in disease burden as assessed by area under the curve (AUC; Fig. 2b), peak disease scores (Fig. 2c) and disease incidence (24/30 [80%] Veh compared to 13/24 [54%] Tet-29; * < 0.05 by Fisher’s exact test). Flow cytometry was used to investigate the impact of Tet-29 on the infiltration of lymphocytes and inflammatory monocytes into the CNS as these immune cells are commonly associated with early disease in EAE [24]. To compare cellular infiltration into the CNS between animals, cell counts in the brain and spinal cord were normalised to microglia as microglia counts did not vary between disease or treatment groups (Additional file 2: Fig S2b). Across both CNS compartments, EAE induced a significant increase in the infiltration of T cells, B cells and Ly6C^high^ monocytes (Fig. 2d–k). In contrast, Tet-29 significantly inhibited the infiltration of CD4^+^ and CD8^+^ T cells into the spinal cord (Fig. 2d, e) and brain (Fig. 2h, i). Tet-29 additionally ameliorated invasion of B cells (Fig. 2j) and inflammatory monocytes (Fig. 2k) into the brain. These effects were not reflected in the periphery as Tet-29 did not alter immune cell counts in the spleen (Fig. 2l–o), although both EAE groups did have slightly reduced CD8^+^ T cell and B cells counts (Fig. 2m, n). Together, these data demonstrates that Tet-29 specifically inhibits CNS infiltration of immune cells in EAE, supporting the amelioration of disease.

**Fig. 3:**
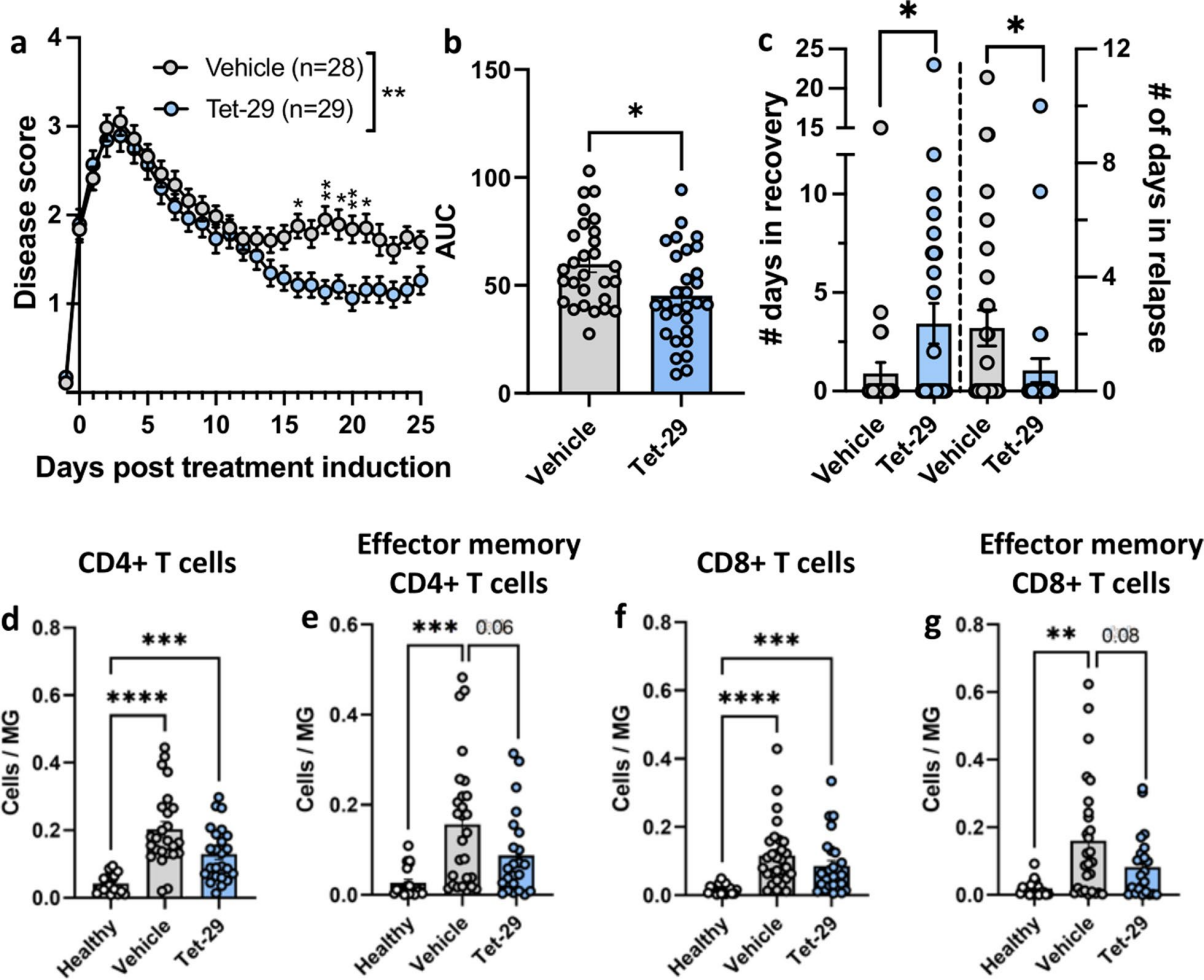
Delivery of Tet-29 after established disease still exerts a disease modifying effect. Female C57Bl/6J mice were immunised for EAE. Treatment with 30 mg/kg/day of Tet-29 or the equivalent volume of vehicle began at disease onset, defined as a disease score of  ≥ 1, and continued for at least 20 days. Mice were scored daily (**a, b**) and days spent in relapse were recorded (**c**). Relapse was defined as an increase in score of  ≥ 0.5 for ≥ 2 days. **d–g** Infiltration of T cells into the spinal cord was investigated by flow cytometry. All data are displayed as mean ± SEM and are pooled from 4 independent experiments, n = 18–28 per group. **a** ****p < 0.0001 by mixed effects ANOVA and *p < 0.05, **p < 0.01 by Sidak’s multiple comparison test. **b, c** *p < 0.05 by Student’s unpaired t-test. **d–g** *p < 0.05, **p < 0.01, ***p < 0.001 by one-way ANOVA with Tukey’s multiple comparisons

Most previous publications on HS-mimetics in EAE report on their use as prophylactic treatments [17] since rescue of disease-induced BBB breakdown is not expected to produce a significant disease-modifying effect after the initial immune cell infiltration. We investigated a more clinically relevant regimen that initiated Tet-29 treatment after disease onset to determine whether therapeutic delivery of an HS-mimetic could reverse CNS infiltration during established disease. Both Tet-29- and vehicle-treated animals followed a similar course of disease for the first 14 days of treatment (Fig. 3a). Like prophylactic treatment, a significant interaction between treatment and time emerged, and Tet-29 significantly reduced the average daily disease scores in the last 10 days of treatment and reduced disease burden as assessed by AUC (Fig. 3b). As therapeutic treatment experiments were carried out over a longer period, the number of animals that recovered from paralysis or experienced a relapse in disease could be evaluated. Tet-29 enhanced disease recovery—both the number (11/29 Tet-29 compared to 4/28 Vehicle; p = 0.07 by Fisher’s exact test) and duration of recovery (Fig. 3c, left) while it significantly decreased the number (4/29 Tet-29 compared to 12/28 Vehicle; p < 0.05) and duration of relapses (Fig. 3c, right). As expected, EAE induced a significant increase in the infiltration of CD4^+^ and CD8+ T cells, as well as their respective CD62L^-^CD44^+^ effector memory subsets, into the spinal cord and brain (Fig. 3d–g; Additional file 3: Fig S3a). Tet-29 treatment did not significantly reduce this EAE-mediated infiltration. However, accumulation of CD4^+^ and CD8^+^ effector memory T cells into the spinal cord of EAE mice treated with Tet-29 appeared to be reduced although the effect did not reach statistical significance. This trend was not observed in the brain (Additional file 4: Fig S4a). Like prophylactic treatment, the effects of Tet-29 on CNS infiltration were not associated with changes in immune cell subsets in the periphery (Additional file 3: Fig S3b). Overall, Tet-29 treatment initiated after established disease could blunt, but not reverse, EAE-induced T cell infiltration into the spinal cord, resulting in a significant reduction in disease symptoms.

### Therapeutic Tet-29 reverses disease-induced blood–brain barrier damage

**Fig. 4:**
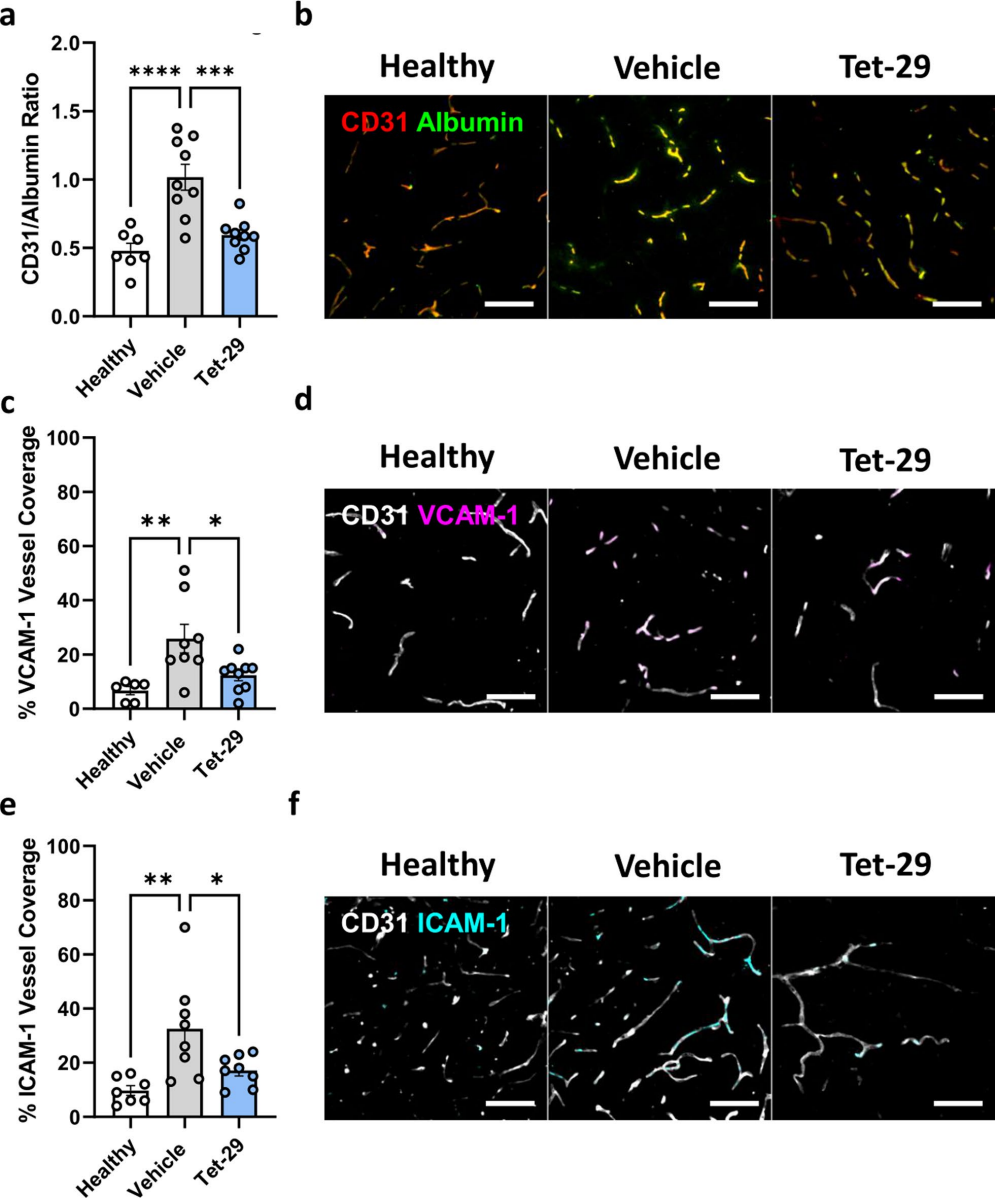
Tet-29 administration returns the inflamed BBB to a phenotype resembling healthy. Brains from female C57Bl/6J mice immunised for EAE and treated daily with 30 mg/kg of Tet-29 from disease onset (disease score ≥ 1) were collected and processed for analysis by confocal microscopy. **a** Blood vessel permeability was quantified by the ratio of CD31 area stained over albumin area stained. **c, e** VCAM-1 and ICAM-1 expression was quantified by the % vessel coverage. **b, d, f** Representative images taken from the cerebellum, blood vessels were visualised with rabbit anti-mouse CD31 (red and grey), and co-stained with either goat anti-mouse albumin (green), rat anti-mouse VCAM-1/CD106 (magenta), or rat anti-mouse ICAM-1/CD54 (cyan). Each data point represents the average of 4–6 regions of interest analysed per section from at least 3 sections per animal with n = 6–9 mice per group. Scalebars = 50 µm. *p < 0.05, **p < 0.01, ***p < 0.001, ****p < 0.0001 by one-way ANOVA with Tukey’s multiple comparisons

To identify whether Tet-29 reduces CNS infiltration in EAE by modulating BBB integrity, the brain vasculature was investigated by confocal microscopy. Albumin staining within the CNS parenchyma is an established method of quantifying long-term changes in blood vessel permeability [25, 26]. In healthy mice, staining for albumin was contained within blood vessels (i.e. co-localisation of albumin and CD31 expression), indicating an intact BBB (Fig. 4b). EAE induced a significant increase in albumin staining outside of blood vessels, as quantified by a ratio of albumin/CD31 coverage, indicating a significant loss of blood vessel integrity. Therapeutic treatment with Tet-29 treatment reversed disease-induced blood vessel permeabilisation, evidenced by a reduction in albumin leakage comparable to healthy controls (Fig. 4a). A similar effect of Tet-29 on BBB integrity was validated by analysing the co-localisation of albumin and Collagen IV expression (Additional file 4: Fig S4a-b).

We next assessed the impact of Tet-29 treatment on the expression of the adhesion molecules VCAM-1 and ICAM-1. Under steady-state conditions, the brain endothelium significantly restricts expression of cell adhesion molecules (CAMs) to regulate cell migration into the parenchyma, whereas upregulation of CAM expression promotes migration across inflamed endothelium [27]. Indeed, we observed a significant increase in VCAM-1 (Fig. 4c, d) and ICAM-1 (Fig. 4e, f) expression in the cerebellum due to EAE, which was reversed in mice treated therapeutically with Tet-29 (Fig. 4c, e). Additionally, when assessing all animals, the expression of VCAM-1 but not ICAM-1 was significantly correlated to the final disease score (p < 0.01 by Sprearman’s r; Additional file 4: Fig S4c). Taken together, analysis of changes at the BBB found that Tet-29 reverses EAE-induced blood vessel permeability and expression of CAMs, suggesting that therapeutic treatment with Tet-29 returns the BBB to a healthy-like phenotype.

### Tet-29 interacts with immune cells via its sulfate groups

**Fig. 5:**
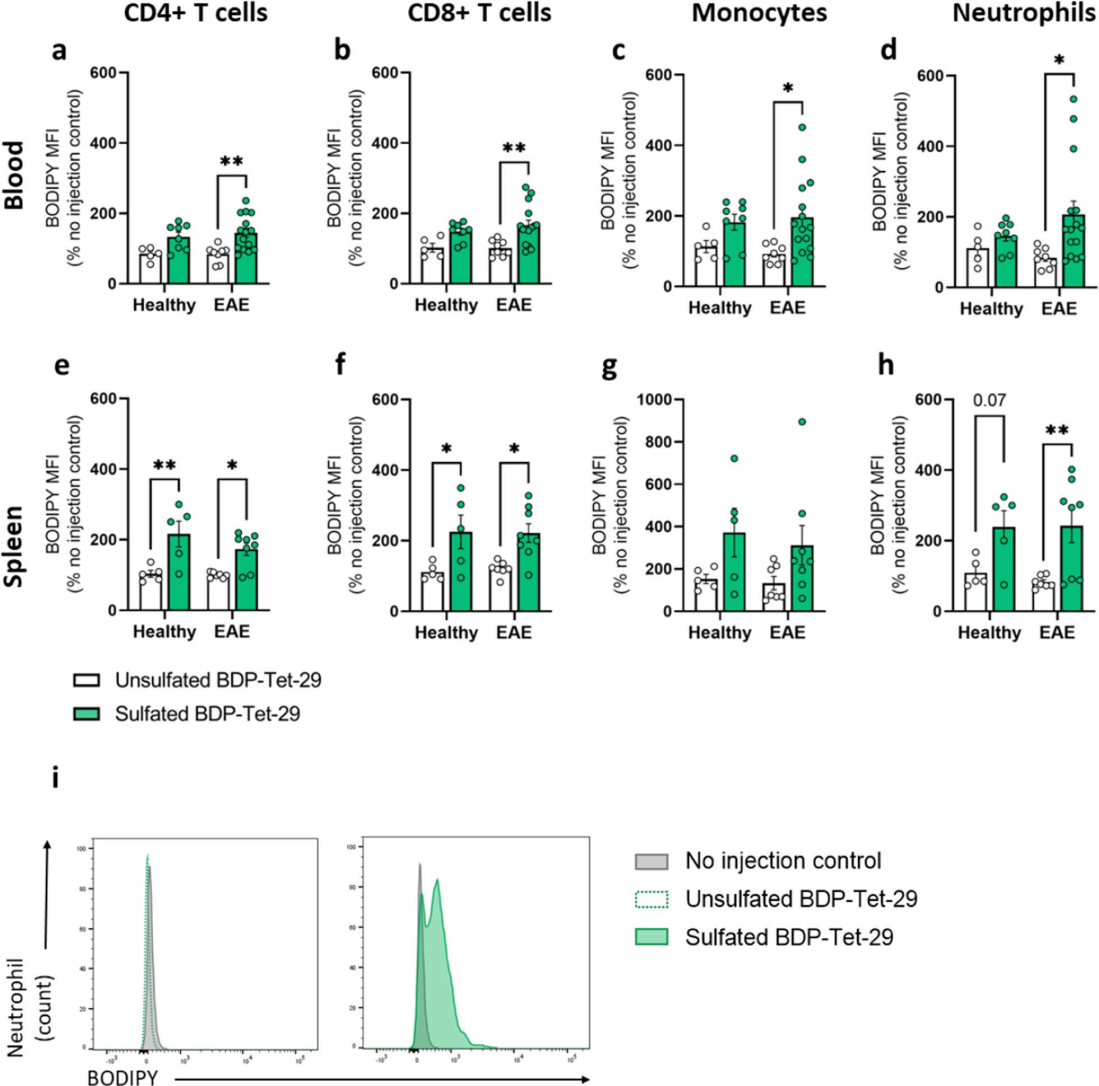
Tet-29 has high affinity for multiple immune cell types. Treatment-naïve healthy and EAE female C57Bl/6J mice were administered a single i.p. dose of BDP-Tet-29 (3 mg/kg; sulfated), unsulfated BDP-Tet-29 (3 mg/kg) or the equivalent volume of PBS (100 µl). The compounds were allowed to circulate for 45 min before tissue was collected and analysed by flow cytometry. BDP expression on immune cells in the blood (**a–d**) and spleen (**e–h**) was quantified by MFI and displayed as a percentage of the vehicle control. Data are pooled from 3 independent experiments and displayed as mean ± SEM with n = 5–15 mice per group, *p < 0.05, **p < 0.01 by two-way ANOVA with Sidak’s multiple comparisons. **i** Representative histograms comparing BDP staining on neutrophils of mice treated with either sulfated BDP-Tet-29 or unsulfated Tet-29 with untreated controls

By reducing the expression of CAMs on brain endothelium, Tet-29 treatment altered the ability of immune cells to interact with the BBB. Next, we investigated whether Tet-29 interacted with immune cells directly, and if this interaction was via the same mechanism as endogenous HS. While Tet-29 is known to bind directly to HPSE [20], it is also likely that Tet-29, as a HS-mimetic, can bind cell surface proteoglycans and other proteins as does endogenous HS [28]. To determine the binding capacity and specificity of Tet-29 in vivo, we administered BODIPY (BDP)-Tet-29, an unsulfated BDP-Tet-29, or vehicle to healthy mice and mice with chronic EAE. Because the sulfate groups on endogenous HS molecules are known to be crucial for its binding capacity and diversity [28], the non-sulfated BDP-Tet-29 served as a negative control. We found that BDP-Tet-29 was able to bind multiple immune cell populations including T cells, monocytes, and neutrophils in the blood of EAE mice (Fig. 5a–d), and BDP-positive cells were also detected in the spleen of healthy and EAE mice (Fig. 5e–h). Unsulfated BDP-Tet-29 could not be detected on immune cells above the level of control animals that received no mimetic (Fig. 5i). These findings indicate that Tet-29 has a strong binding affinity for immune cells in vivo*,* and this interaction is dependent on its sulfate groups.

### Tet-29 inhibits inflammatory T cell trafficking across CNS barrier models in vitro

**Fig. 6:**
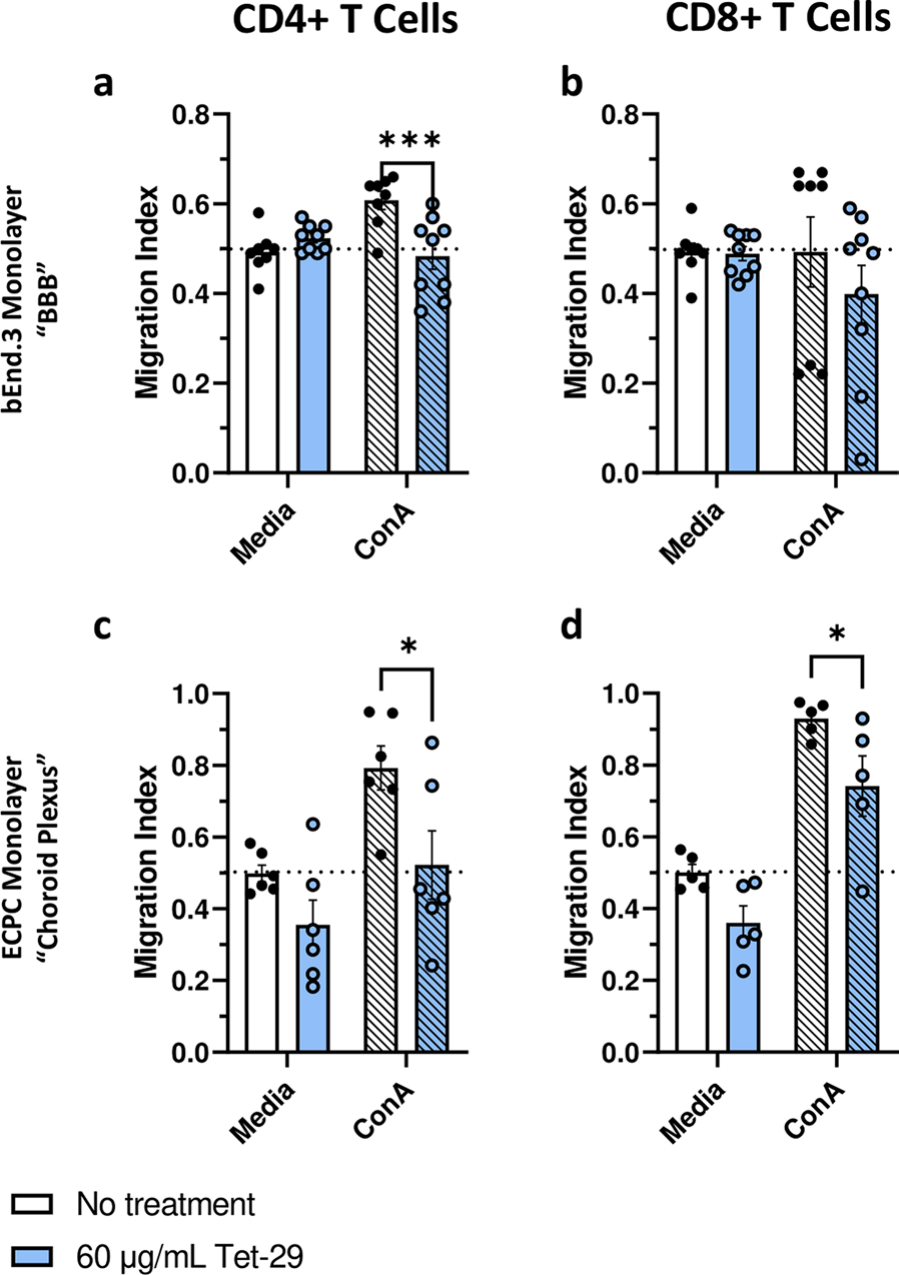
Tet-29 inhibits inflammatory T cell migration in in vitro barrier models. In vitro models of the ChP and BBB. Splenocytes stimulated with ConA (1 μg/mL) for 24 h were co-treated ± Tet-29 at 6 μg/mL for 48 h and then added to the upper well of a Transwell culture seeded with a monolayer of endothelial **(a, b)** or epithelial cells **(c, d)**. Transwell cultures were incubated for 24 h, and the extent of lymphocyte migration was assessed by flow cytometry of the upper and lower chambers. Data are pooled from 3 independent experiments and displayed as mean ± SEM with n = 5–8 biological replicates per group, *p < 0.05, ***p < 0.001 by two-way ANOVA with Sidak’s multiple comparisons

To investigate the barrier specific effects of Tet-29 on immune cell migration, we utilised an in vitro monolayer model of the BBB (inflammatory migration) and the choroid plexus (ChP; inflammatory and homeostatic trafficking.) Across both the ChP and BBB models, ConA pre-stimulation induced a significant increase in the number of CD4^+^ and CD8^+ ^T cells trafficking into the “CNS” compartment although CD8^+^ trafficking across the bEnd.3 monolayer was unaffected (Fig. 6a–d). This effect was more prominent across the epithelial versus endothelial barrier, which is consistent with the in vivo characteristics of these barriers, whereby the ChP is more permeable to immune cell diapedesis than the BBB [29]. An increase in T cell trafficking is characteristic of MS, in which relapses are predominantly mediated by aberrant and unregulated pro-inflammatory T cell trafficking into the neural parenchyma [30]. In our endothelial model of the BBB, Tet-29 abolished ConA mediated trafficking of CD4^+^ (Fig. 6a), whereas Tet-29 significantly reduced inflammatory trafficking of both CD4^+^ and CD8^+^ T cells across the epithelial ChP model (Fig. 6c, d). Interestingly, Tet-29 did not significantly alter trafficking across either barrier in unstimulated conditions. Together, these results suggest that Tet-29 inhibits inflammatory but not homeostatic T cell trafficking into the CNS across the ChP and BBB.

### Tet-29 does not inhibit CNS infiltration under homeostatic conditions

To assess the impact of Tet-29 on homeostatic CNS trafficking, healthy mice were treated with daily Tet-29 (30 mg/kg), PI-88 (10 mg/kg), or vehicle, or every 4 days with an anti-VLA-4 monoclonal antibody (αVLA-4; PS/2; 5 mg/kg) for a minimum of 10 days. PI-88 (Muparfostat; Additional file 1: Fig S1) is an HS-mimetic developed as an angiogenesis and tumour metastasis inhibitor [16]. Like Tet-29, PI-88 exhibits a beneficial effect in EAE and demyelinating models [14, 31]. The 10 mg/kg/day dose used in this study has previously been used in a chronic autoimmune model of diabetes in mice, where it significantly delayed the onset of disease [12]. The effect of Tet-29 was also compared to αVLA-4, a murine version of natalizumab, as this is a known inhibitor of homeostatic trafficking into the CNS of humans [32]. By binding VLA-4 (Additional file 5: Fig S5a-b), αVLA-4 prevents the formation of an adhesion complex with VCAM-1 and effectively reduces leukocyte accumulation in the CNS [33]. The 5 mg/kg dose every 4 days was selected based on previous literature reporting a beneficial effect of αVLA-4 treatment in EAE when treatment is initiated before the onset of disease [34, 35].

**Fig. 7:**
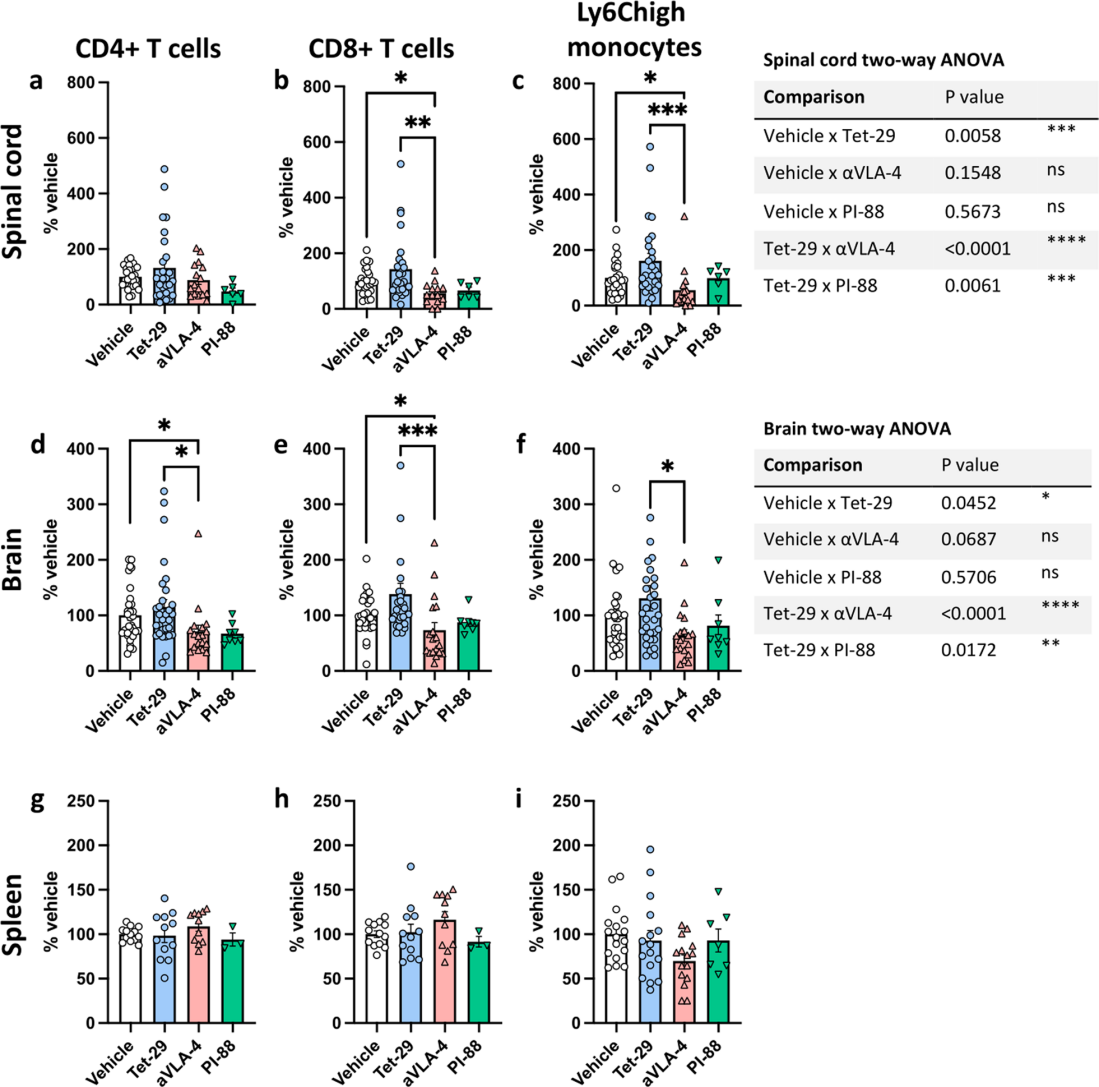
Tet-29 does not inhibit homeostatic CNS infiltration. Healthy, female, C57Bl/6J mice were treated with 30 mg/kg/day of Tet-29, 10 mg/kg/day of PI-88, or 5 mg/kg of αVLA4 monoclonal antibody every four days for 10–23 days total. At the end of treatment, the distribution of leukocytes in spinal cord (**a–c**), brain (**d–f**), and spleen (**g–i**) was analysed by flow cytometry. Data were pooled from up to 8 independent experiments and displayed as mean ± SEM (n = 3–32 per group; full details in Additional file 6: Fig S6). *p < 0.05, **p < 0.01, ***p < 0.001 by Kruskal–Wallis with Dunn’s multiple comparison test (**a–i**) and by two-way ANOVA with Tukey’s multiple comparisons to assess the overall effect of treatments (right-hand tables)

Mice treated with αVLA-4 exhibited significantly lower numbers of CD8^+^ T cells and Ly6C^high^ monocytes in the spinal cord and brain compared to Tet-29-treated animals while CD4^+^ T cell trafficking was reduced in the brain but unchanged in the spinal cord (Fig. 7a–f). Compared to vehicle, αVLA-4 also significantly lowed the number of CD8+ T cells and Ly6C^high^ monocytes in the spinal cord and CD8^+^ and CD4^+^ T cells in the brain, and hese findings are consistent with previous reports [36, 37]. Neither the Tet-29 nor the PI-88 treatment groups deviated significantly from vehicle alone when the individual cell types were compared. However, we found that Tet-29 treatment had a significant overall affect with increased CNS infiltration in the brain and spinal cord of Tet-29-treated animals compared to vehicle, αVLA-4, and PI-88 (2-way ANOVA; Fig. 7). In comparison to EAE-induced migration where there was an increase in immune cells (e.g. 5.8-fold for CD8^+^ T cells in the brain; Fig. 2i), Tet-29 treatment in healthy animals increased homeostatic trafficking compared to vehicle (e.g. 1.38 fold for CD8^+^ T cells in the brain; Fig. 7e). Interestingly, the effect of Tet-29 on homeostatic migration appeared specific, as PI-88 did not follow the same trend and was significantly different from Tet-29 in the brain and spinal cord (Fig. 7). As expected, the impact of these treatments on CNS invasion was not reflected in the periphery as no treatment group exhibited significant differences in cell frequencies in the spleen (Fig. 7g–i). Taken together, although the effects on homeostatic CNS trafficking are modest, these data indicate that Tet-29 may be less disruptive to this trafficking pathway than αVLA-4 and is distinctly different from PI-88.

## Discussion

This study investigated the disease-modifying effect of Tet-29 in EAE. Recent studies have found that low sulfated HS-mimetics can ameliorate disease by sequestering proinflammatory chemokines in MS disease models [18]. Here, we focused on whether Tet-29 could alter immune cell migration across barriers of the CNS. We found that Tet-29 significantly reduced inflammatory infiltration of leukocytes into the CNS in vivo and prevented migration across brain endothelial and epithelial barriers in vitro. Moreover, Tet-29 elicited a modest but significant positive effect on migration under steady-state conditions. This work provides evidence that HS-mimetics could be effective therapeutics in neuroinflammatory conditions like MS, potentially without disrupting homeostatic immune surveillance.

Previous studies have reported therapeutic benefits of HS-mimetics in EAE delivered both before [15, 17] and after [18] the onset of disease and that similar HS-mimetics could be orally delivered [18]. In concordance with these reports, we found that Tet-29 delivered both before and after the onset of disease significantly ameliorated symptoms. Administration before symptom onset significantly reduced disease incidence, suggesting that it interfered with the initial CNS infiltration of myelin-specific T cells [38, 39]. A similar mechanism could explain the reduced relapse rate in animals that were treated after established disease, as relapses of physical symptoms follow the infiltration of myelin-specific memory T cells that persist in the periphery [40]. The effect of Tet-29 on the EAE pattern of disease led us to investigate the impact of this HS-mimetic on the CNS infiltration of different leukocyte subsets.

A key feature of EAE is CNS infiltration of predominantly T cells and inflammatory Ly6C^high^ monocytes [24, 41]. Prophylactic treatment with Tet-29 reduced these lymphocyte and myeloid cell types in the CNS, indicating a generalised reduction in neuroinflammation, which has been reported with other HS-mimetics [18]. In comparison, therapeutic treatment with Tet-29 elicited a reduction in T cell accumulation, particularly of CD44^+^ CD62L^−^ effector memory T cells, in the CNS. The specific inhibition of effector T cell infiltration in EAE has also been observed with the HS-mimetic PG545 [17]. This shared effect may be due to the ability of both HS-mimetics to inhibit HPSE activity [20]. Activated T cells are known to produce high levels of HPSE, which correlates with their ability to penetrate blood vessels and, in turn, their encephalitogenic potential in EAE [8, 9, 42]. By mainly targeting effector T cell infiltration, Tet-29 specifically inhibits CNS infiltration of those cells that drive both induction of disease and subsequent relapses [43, 44]. Tet-29 treatment did not affect the number of T cells in peripheral lymphoid organs such as the spleen, suggesting that its effect on T cell accumulation in the CNS is due to reduced migration across the BBB, rather than systemic depletion of these cells.

We hypothesised that therapeutic Tet-29 blunted T cell infiltration into the CNS by inhibiting the HPSE-mediated breakdown of HSPGs at the BBB. During EAE, CNS blood vessels exhibit increased permeability to serum proteins, which correlates to CNS leukocyte accumulation and more severe disease symptoms [45, 46]. Furthermore, increased blood vessel permeability during inflammation has been attributed to elevated leukocyte- and endothelial-derived HPSE in models of delayed-type hypersensitivity [10]. We found that EAE mice treated therapeutically with Tet-29 exhibited significantly lower CNS blood vessel permeability to albumin compared to mice treated with vehicle alone, indicating a reversal of EAE-induced BBB permeabilisation. BBB permeability measured by albumin leakage represents diffuse long-term changes over the course of the disease rather than focal disruption due to inflammation [25, 45]. Therefore, it was important to also investigate the impact of Tet-29 on adhesion markers, which are upregulated during inflammation. Therapeutic treatment with Tet-29 reversed the disease-induced increase in VCAM-1 and ICAM-1 expression on brain endothelium. Both VCAM-1 and ICAM-1 are upregulated during inflammation and are essential for T cell arrest and diapedesis across brain endothelium [47]. It is possible that the reduction in adhesion marker expression is caused by an overall decrease in inflammatory milieu surrounding brain endothelial cells in Tet-29-treated mice. The reduced CAM expression in Tet-29 treated animals is consistent with the inverse relationship between CAM and HSPG expression observed in the literature. Inflammation-induced damage to the vascular glycocalyx (including HSPGs) exposes VCAM-1 and ICAM-1, promoting further adherence of circulating leukocytes to endothelium and propagating more inflammation [48]. This is supported by gene knockout studies that find mice lacking syndecan-1 exhibit increased ICAM-1 expression in DTH models, which enhances leukocyte-endothelial interactions [49]. Our investigations into BBB permeability and CAM expression showed that Tet-29 treatment rescues the integrity of the BBB, suggesting that steady-state functioning should not be impacted by Tet-29 treatment.

During EAE and MS, leukocytes invade the CNS predominantly by migrating across the endothelial BBB [50]. However, an often-overlooked route of entry is via the ChP. T cell trafficking across the epithelial barrier of the ChP is essential for the development of EAE [38] and has a critical role in immune surveillance of the CNS [51]. In monolayer models of the BBB and the ChP, we observed a higher migration index in the ChP model compared to the BBB model. While these models are less complex than the in vivo situation, our findings are consistent with the higher permeability of the ChP in comparison to the BBB in vivo, due to fenestrations and constitutive expression of adhesion markers for cell trafficking in the epithelial cell barrier [52] and also supports their use as models of in vivo barriers for both inflammatory and steady-state trafficking. In both models, Tet-29 inhibited migration of T cells in ConA-stimulated, but not unstimulated, conditions, suggesting that the modulation of CNS trafficking by Tet-29 is dependent on the inflammatory environment. If the effects of Tet-29 in EAE are primarily due to its ability to inhibit HPSE, the difference between the effects on ConA-stimulated and unstimulated migration could be due to the low endoglycosidase activity of HPSE at a physiological pH under normal conditions [53].

In non-inflammatory conditions, HPSE expression by endothelial, epithelial, and circulating leukocytes is low [54–56] suggesting that Tet-29 would have minimal impact on migration. However, unlike its effect in EAE, Tet-29 had no negative impact on cellular migration into the CNS in healthy mice and appeared to subtly enhance migration. In contrast, another HS-mimetic, PI-88, did not show this enhancement and had minimal impact on homeostatic CNS trafficking. We speculate that homeostatic migration is supported during Tet-29 treatment as this trafficking occurs primarily across the ChP, which does not require HPSE-dependent ECM remodelling [29, 57]. Therefore, homeostatic trafficking would not be negatively impacted by changes in HPSE expression or activity.

Compared to Tet-29, treatment with αVLA-4 significantly reduced CD8^+^ T cell, CD4^+^ T cell and Ly6C^high^ monocyte accumulation in the CNS. This finding is consistent with the disrupted CNS homeostatic trafficking that occurs in people with MS treated with the monoclonal antibody to VLA-4, natalizumab [58]. The ChP constitutively expresses the adhesion molecules VCAM-1, ICAM-1 and p-selectin [29], whereas CAM expression at the BBB is upregulated in response to inflammatory signals [59]. Therefore, preventing VLA-4/VCAM-1 adhesion with natalizumab inhibits both homeostatic and inflammatory trafficking into the CNS [58]. Complete abolishment of CNS immune surveillance has been implicated in the development of progressive multifocal leukoencephalopathy (PML) in people with MS treated with natalizumab [60]. While PML is rare, its risk means that people with MS require additional screening steps prior to natalizumab treatment and, in turn, excludes some people from an otherwise effective treatment [61]. Again, while we see that Tet-29 returns VCAM-1 expression to normal during neuroinflammation, this does not appear to impair VCAM-1 mediated migration across the ChP under homeostatic conditions. Our group is currently investigating Tet-29-induced changes to adhesion markers at the ChP. For now, this work highlights the potential to develop MS treatments that more directly target inflammatory and not homeostatic leukocyte migration into the CNS. Our results demonstrate that inhibition of trafficking via the maintenance of barriers of the CNS, rather than by blocking adhesion molecules, can limit but not completely restrict access to the CNS. In the future, head-to-head comparisons will enable selection of therapeutics for individual patients that balance clinical efficacy and the risk of adverse events.

Finally, we investigated the distribution of fluorescently labelled Tet-29 in vivo. Unsulfated Tet-29 did not bind leukocytes, indicating that, like endogenous HS, Tet-29 exerts its function via its sulfate groups [28]. The similarity to endogenous HS suggests that Tet-29 has the potential to bind many ligands, including growth factors, cytokines, chemokine receptors and integrins [62]. Lindsay and colleagues [18] identified several chemokines involved in EAE pathogenesis that bind the low sulphated HS-mimetic LS-mHep7. Work from our group is currently underway to identify chemokines that are targeted by Tet-29. This will help deduce how chemokine gradients, binding availability, and dimerization may contribute to the opposing effect of Tet-29 on homeostatic versus inflammatory trafficking.

## Conclusion

Tet-29 has exhibited promising disease-modifying effects in myeloma and colorectal cancer models [20, 63]. The data presented here supports further investigation into Tet-29 as a potential treatment for neuroinflammatory disorders like MS. Tet-29 is particularly promising as a therapeutic in autoimmune-mediated CNS inflammation because of its ability to inhibit inflammatory trafficking whilst supporting steady-state CNS migration.

### Supplementary Information

**Additional file 1: Figure S1.** The chemical structure of PI-88.

**Additional file 2: Figure S2.** Gating strategies for flow cytometry. (a) Cells of interest were identified by forward scatter (FSC) and side scatter (SSC) parameters, and CD45 expression. (b) Microglia and leukocytes were differentiated based on their CD45 expression, and there was no significant difference in microglia counts in the brain between disease or treatment groups. Data are pooled from 4 independent experiments and displayed as mean ± SEM (n = 12–18 per group). (c) Gating strategy to identify lymphocytes from single-cell, CD45^+^ populations. (d) Gating strategy to identify myeloid cells from single-cell, CD45^+^ populations.

**Additional file 3:** **Figure S3.** Therapeutic Tet-29 treatment had minimal impact on brain or spleen T cell populations. Immune cell populations in the brain (a) and spleen (b) of mice treated therapeutically with Tet-29 were analysed. Data are displayed as mean ± SEM and are pooled from 4 independent experiments with n = 18–28 per group. *p < 0.05, **p < 0.01, ***p < 0.001 by one-way ANOVA with Tukey’s multiple comparisons.

**Additional file 4: Figure S4.** Tet-29 reduces BBB permeability when blood vessels are visualised with collagen IV staining. Brains from female C57Bl/6J mice immunised for EAE and treated daily with 30 mg/kg of Tet-29 from disease onset (disease score ≥ 1) were collected and processed for analysis by confocal microscopy. Brains were stained with albumin (green) and collagen IV (red). (a) Blood vessel permeability was quantified by the ratio of collagen IV area stained over albumin area stained. Each data point represents the average of 4 regions of interest analysed per section from 1–2 sections per animal with n = 6–10 mice per group. *p < 0.05 by one-way ANOVA with Tukey’s multiple comparisons. (b) Representative taken from the cerebellum of healthy and vehicle- or Tet-29-treated EAE mice. Scalebar = 100 µm. (c) VCAM-1 or ICAM-1 expression and blood vessel permeability for individual animals are compared to the final score and total score (i.e. area under the curve; AUC), respectively and as described in Fig. 4.

**Additional file 5: Figure S5.** αVLA-4 administration blocks VLA-4 expression on circulating leukocytes. Healthy, female, C57Bl/6J mice were treated with vehicle, 5 mg/kg of αVLA4, or 5 mg/kg of an isotype control (rat IgG2b anti-keyhole limpet hemocyanin) every four days for 10–23 days total. VLA-4 (CD49d) expression was analysed by flow cytometry in lymphocytes (a) and Ly6C^+^ monocytes (b). Data are displayed as mean ± SEM with n = 4 replicates per group. **p < 0.01, ***p < 0.001, ****p < 0.0001 by two-way ANOVA with Tukey’s multiple comparisons.

**Additional file 6: Figure S6.** Number of animals and independent experiments for Fig. 7.

## Data Availability

The datasets used and/or analysed during the current study are available from the corresponding author on reasonable request.

## Abbreviations

BBB: Blood brain barrier
BODIPY: 4,4-Difluoro-4-bora-3a,4a-diaza-*s*-indacene
ChP: Choroid plexus
CNS: Central nervous system
ConA: Concanavalin A
EAE: Experimental autoimmune encephalomyelitis
ECM: Extracellular matrix
ECPC-4: Epithelial choroid plexus cell line-4
FACS: Flow cytometry staining buffer
FCS: Foetal calf serum
HPSE: Heparanase
HS: Heparan sulfate
HSPG: Heparan sulfate proteoglycan
ICAM: Intercellular adhesion molecule
MS: Multiple sclerosis
PBS: Phosphate buffered saline
VCAM: Vascular cell adhesion molecule
