# Supplementary figures and images for: The novel HS-mimetic, Tet-29, regulates immune cell trafficking across barriers of the CNS during inflammation
Source: J Neuroinflammation. 2023 Nov 1;20:251. doi: 10.1186/s12974-023-02925-4 (PMC10619265; doi:10.1186/s12974-023-02925-4)

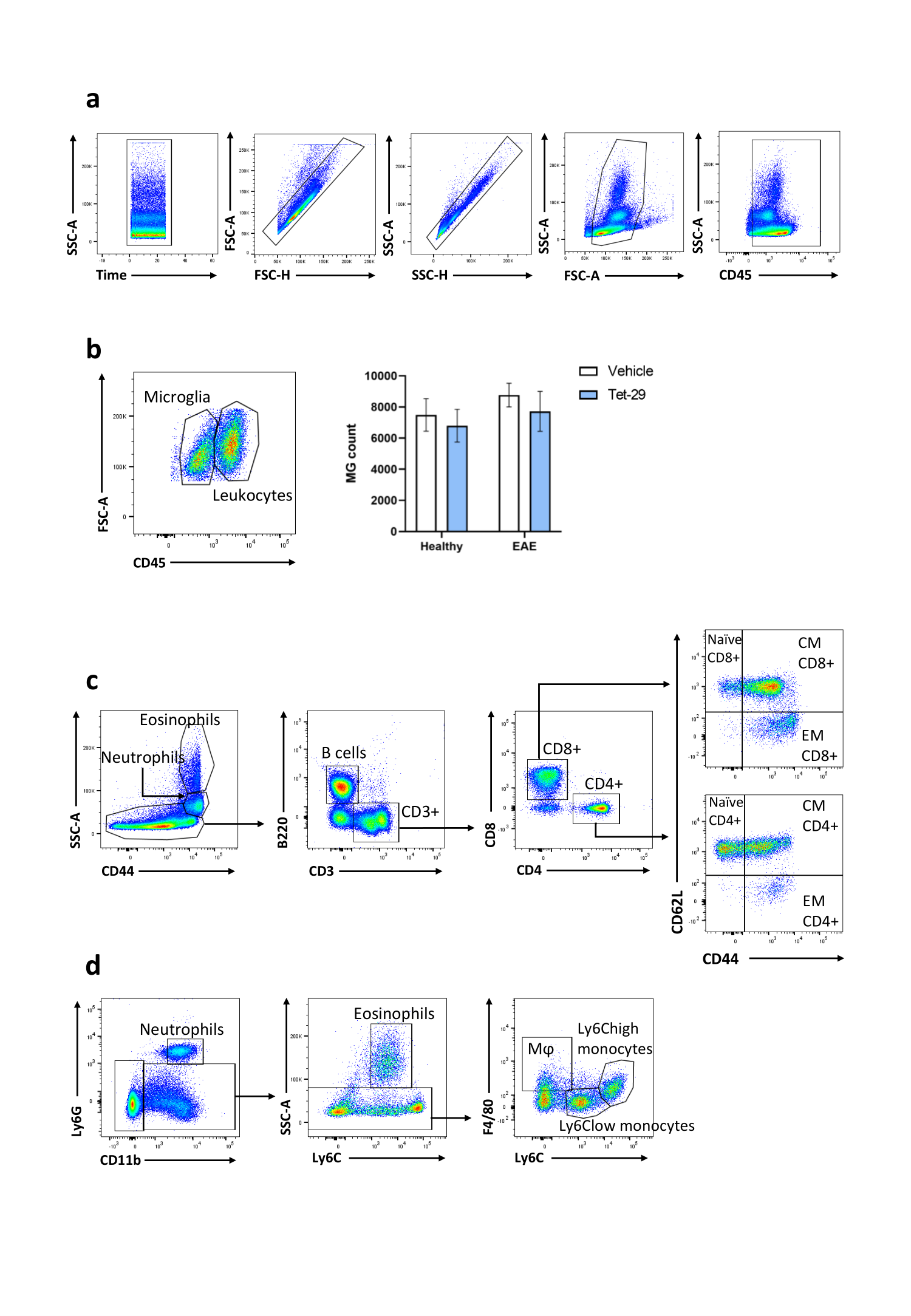

Supplement: Supplementary file 2 — Additional file 2: Figure S2. Gating strategies for flow cytometry. (a) Cells of interest were identified by forward scatter (FSC) and side scatter (SSC) parameters, and CD45 expression. (b) Microglia and leukocytes were differentiated based on their CD45 expression, and there was no significant difference in microglia counts in the brain between disease or treatment groups. Data are pooled from 4 independent experiments and displayed as mean ± SEM (n = 12–18 per group). (c) Gating strategy to identify lymphocytes from single-cell, CD45+ populations. (d) Gating strategy to identify myeloid cells from single-cell, CD45+ populations. [file 12974_2023_2925_MOESM2_ESM.tiff]

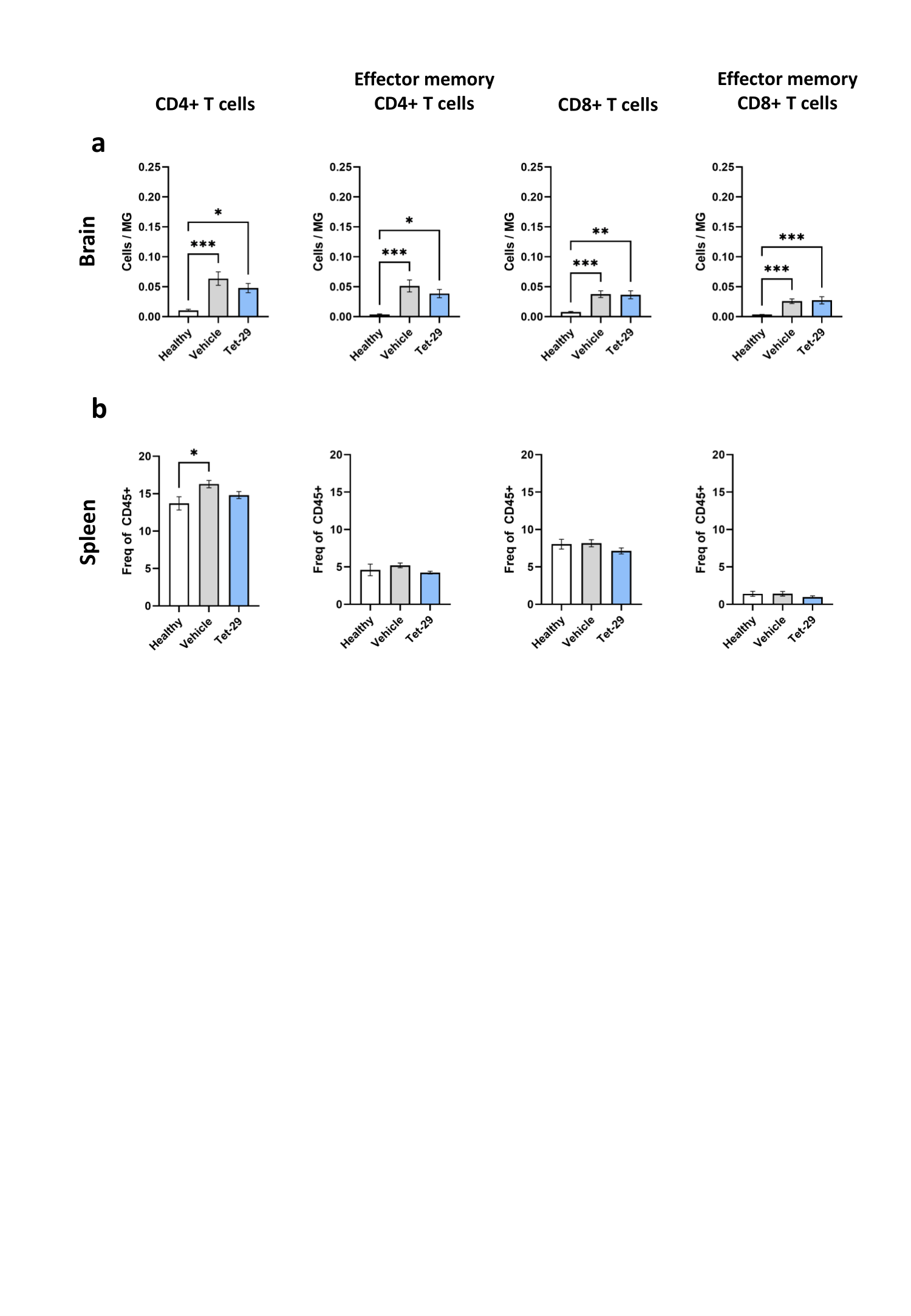

Supplement: Supplementary file 3 — Additional file 3: Figure S3. Therapeutic Tet-29 treatment had minimal impact on brain or spleen T cell populations. Immune cell populations in the brain (a) and spleen (b) of mice treated therapeutically with Tet-29 were analysed. Data are displayed as mean ± SEM and are pooled from 4 independent experiments with n = 18–28 per group. *p < 0.05, **p < 0.01, ***p < 0.001 by one-way ANOVA with Tukey’s multiple comparisons. [file 12974_2023_2925_MOESM3_ESM.tiff]

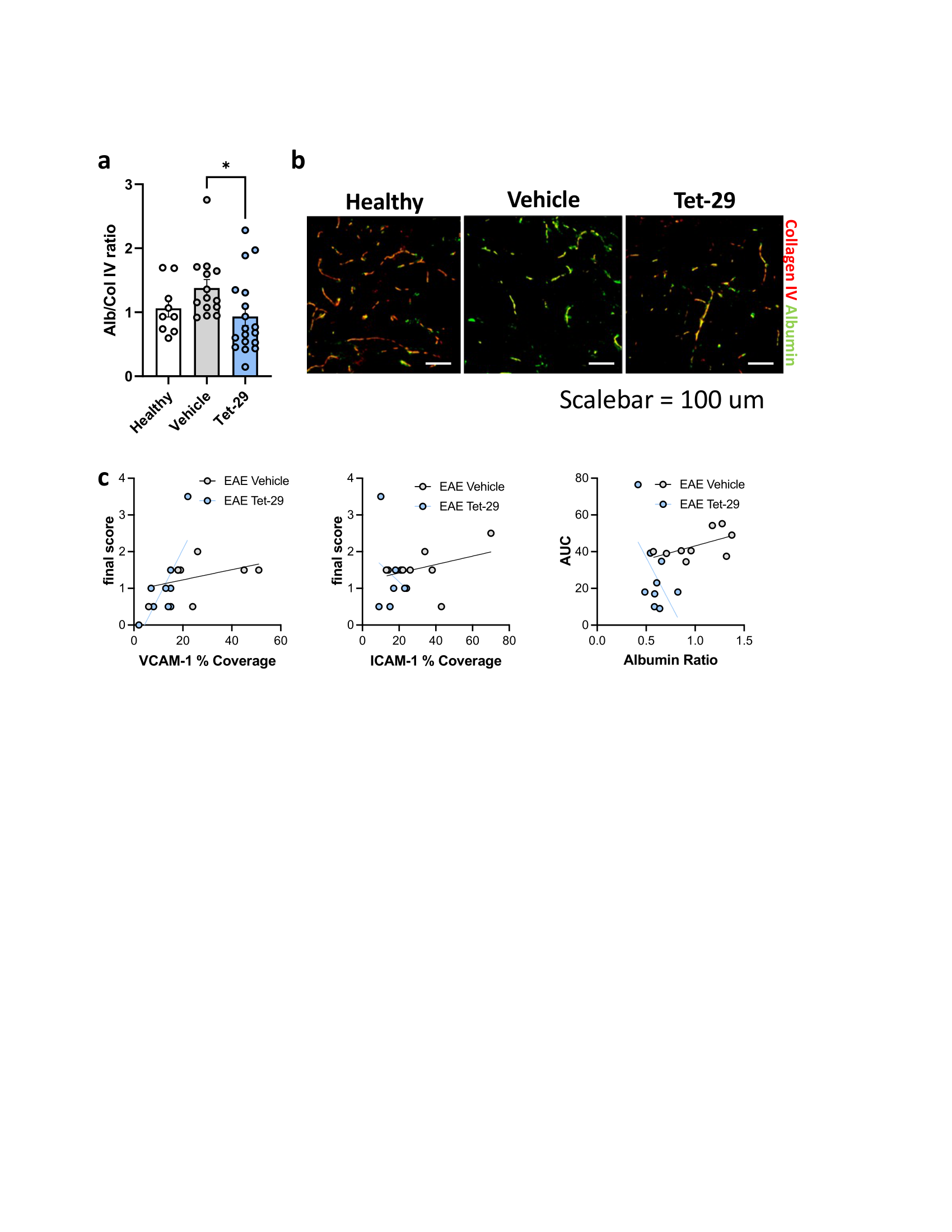

Supplement: Supplementary file 4 — Additional file 4: Figure S4. Tet-29 reduces BBB permeability when blood vessels are visualised with collagen IV staining. Brains from female C57Bl/6J mice immunised for EAE and treated daily with 30 mg/kg of Tet-29 from disease onset (disease score ≥ 1) were collected and processed for analysis by confocal microscopy. Brains were stained with albumin (green) and collagen IV (red). (a) Blood vessel permeability was quantified by the ratio of collagen IV area stained over albumin area stained. Each data point represents the average of 4 regions of interest analysed per section from 1–2 sections per animal with n = 6–10 mice per group. *p < 0.05 by one-way ANOVA with Tukey’s multiple comparisons. (b) Representative taken from the cerebellum of healthy and vehicle- or Tet-29-treated EAE mice. Scalebar = 100 µm. (c) VCAM-1 or ICAM-1 expression and blood vessel permeability for individual animals are compared to the final score and total score (i.e. area under the curve; AUC), respectively and as described in Fig. 4. [file 12974_2023_2925_MOESM4_ESM.tiff]

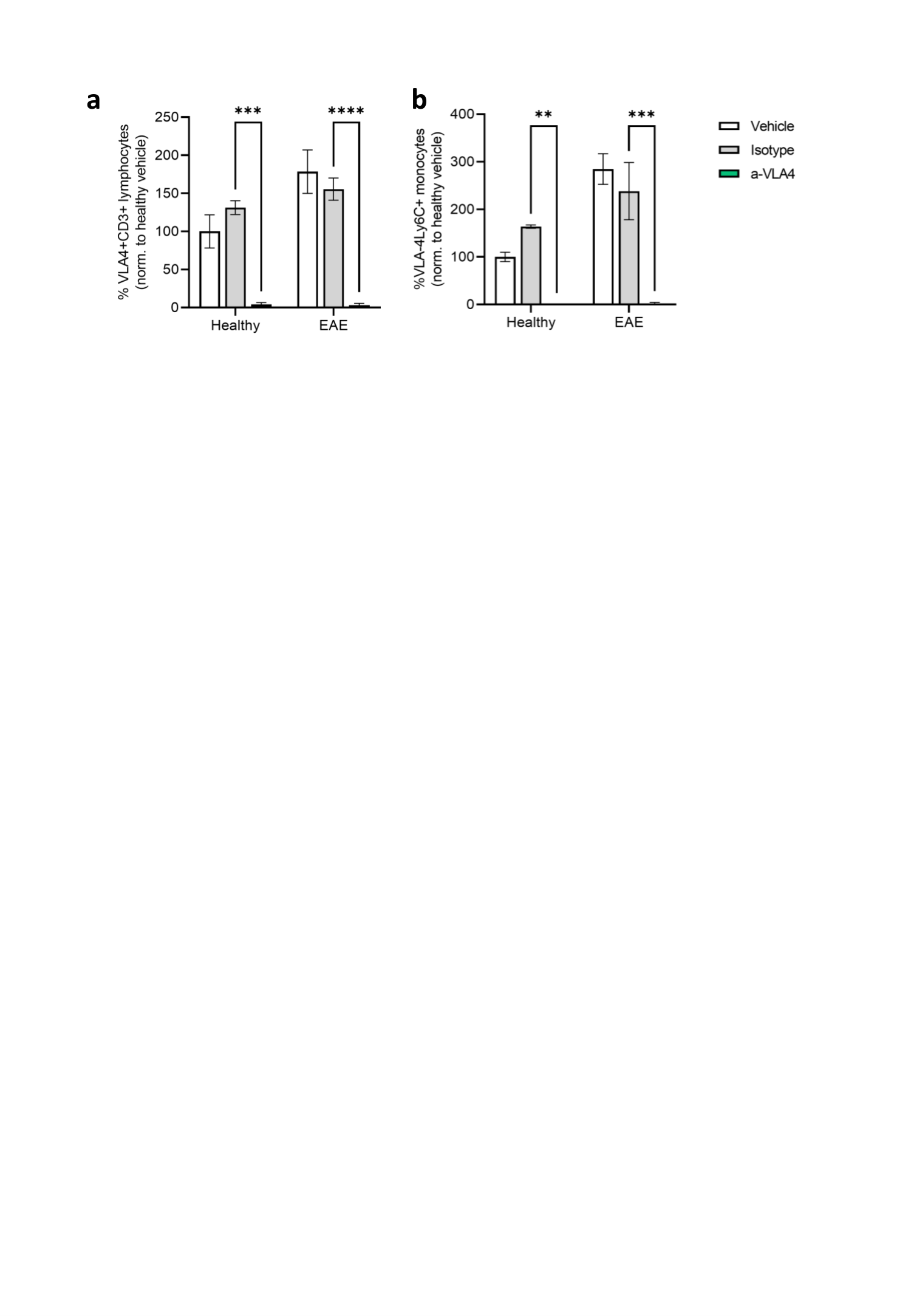

Supplement: Supplementary file 5 — Additional file 5: Figure S5. αVLA-4 administration blocks VLA-4 expression on circulating leukocytes. Healthy, female, C57Bl/6J mice were treated with vehicle, 5 mg/kg of αVLA4, or 5 mg/kg of an isotype control (rat IgG2b anti-keyhole limpet hemocyanin) every four days for 10–23 days total. VLA-4 (CD49d) expression was analysed by flow cytometry in lymphocytes (a) and Ly6C+ monocytes (b). Data are displayed as mean ± SEM with n = 4 replicates per group. **p < 0.01, ***p < 0.001, ****p < 0.0001 by two-way ANOVA with Tukey’s multiple comparisons. [file 12974_2023_2925_MOESM5_ESM.tiff]
